# Activation of Endothelial Pro-resolving Anti-Inflammatory Pathways by Circulating Microvesicles from Non-muscular Myosin Light Chain Kinase-Deficient Mice

**DOI:** 10.3389/fphar.2016.00322

**Published:** 2016-09-21

**Authors:** Abderahim Gaceb, Luisa Vergori, M. C. Martinez, Ramaroson Andriantsitohaina

**Affiliations:** ^1^INSERM U1063, Université d’AngersAngers, France; ^2^Centre Hospitalo-Universitaire d’AngersAngers, France

**Keywords:** endothelial cells, extracellular vesicles, lipopolysaccharide, nmMLCK

## Abstract

Microvesicles, small membrane vesicles released from cells, have beneficial and/or deleterious effects in sepsis. We previously reported that non-muscle myosin light chain kinase (nmMLCK) deletion protects mice against endotoxic shock by reducing inflammation. Here, we have evaluated the consequences of nmMLCK deletion on microvesicle phenotypes and their effects on mouse aortic endothelial cells in association with vascular inflammation and endothelial dysfunction during endotoxic shock induced by lipopolysaccharide in mice. Treatment with lipopolysaccharide induced an increase in levels of circulating microvesicles in wild type but not in nmMLCK-deficient mice. Microvesicles from nmMLCK-deficient mice (MVs^nmMLCK-/-^) prevented the inflammatory effects of lipopolysaccharide with concomitant increase of anti- inflammatory and reduction of pro-inflammatory secretome in mouse aortic endothelial cells. In addition, MVs^nmMLCK-/-^ reduced the efficacy of lipopolysaccharide to increase aortic oxidative and nitrosative stresses as well as macrophage infiltration in the aorta. Moreover, MVs^nmMLCK-/-^ prevented *ex vivo* endothelial dysfunction, vascular hyporeactivity, and *in vivo* overproduction of nitric oxide in heart and liver in response to lipopolysaccharide. Altogether, these findings provide evidence that nmMLCK deletion generates circulating microvesicles displaying protective effects by activating endothelial pro-resolving anti-inflammatory pathways allowing the effective down-regulation of oxidative and nitrative stresses associated with endotoxic shock. Thus, nmMLCK plays a pivotal role in susceptibility to sepsis via the control of cellular activation and release of circulating microvesicles.

## Introduction

Sepsis is defined as a clinical syndrome characterized by a systemic inflammatory response to infection that leads to microvascular thrombosis, vascular hyporeactivity, and endothelial dysfunction resulting in multiple organ dysfunction (Annane et al., 2005; Schouten et al., 2008). Treatment of sepsis is still a clinical challenge in cardiovascular medicine. Among the potential new targets for sepsis therapy, a new protein has been found to play an important role during endotoxic shock in different experimental models of sepsis (Ralay Ranaivo et al., 2007). This protein, the nmMLCK, belongs to a family of protein kinases whose main function is to phosphorylate the 20 kDa regulatory MLC-2 at Ser-19 for ATPase driven actin-myosin contraction. nmMLCK is expressed in endothelial cells, epithelium, platelets, and neutrophils (Verin et al., 1998; Signorello et al., 2013). nmMLCK plays a significant role in the maintenance of endothelial barrier function by controlling the permeability of tight junctions and leukocyte transmigration (Ma et al., 2005; Vandenbroucke et al., 2008). Previously, we showed that nmMLCK knockout mice have lower susceptibility to septic injury and these mice present an improving of survival curve, protection against vascular hyporeactivity, as well as nitrative and oxidative stress associated to endotoxic shock, suggesting a protective role of nmMLCK deletion (Ralay Ranaivo et al., 2007).

Systemic inflammation is orchestrated by interactions between inflammatory cells and target cells by various means of cellular communication such as MVs. MVs represent a circulating reservoir of bioactive molecules displaying multiple functions (coagulation, fibrinolysis, inflammation, and angiogenesis) which are able to carry biological information. Enhanced levels of circulating MVs from platelets, granulocytes, and endothelial cells have been described in patients with meningococcal septicemia, and septic shock (Nieuwland et al., 2000; Fujimi et al., 2002; Mostefai et al., 2008). MVs from septic shock patients exert pleiotropic and differential effects. Indeed, platelet- and leukocyte-derived MVs have procoagulant effects with thrombin generation occurring via a tissue factor VIIa-dependent pathway; this may account to microvascular thrombosis in septic patients (Nieuwland et al., 2000). Accordingly with this, inoculation of septic MVs in healthy rats reproduced hemodynamic, septic inflammatory patterns, associated with oxidative and nitrosative stresses (Mortaza et al., 2009). Conversely, we have demonstrated that MVs possess a protective effect at the early phase of septic shock in humans; thereby protecting against vascular hyporeactivity (Mostefai et al., 2008). Besides, Soriano et al. (2005) have shown that elevated levels of circulating MVs negatively correlated with sequential organ failure assessment score and with survival of septic patients. Altogether, these reports suggest that, in sepsis, MVs are effectors that participate in the pathogenesis and outcome of the disease.

In the present study, we have evaluated the consequences of nmMLCK deletion on MV phenotypes and their effects on secretome of mouse aortic endothelial cells. Also, we have analyzed their effects on vascular inflammation and endothelial dysfunction subsequent to endotoxic shock induced by lipopolysaccharide (LPS) in mice. For this, by using MVs^nmMLCK+/+^ and MVs^nmMLCK-/-^ in a model of cellular and *in vivo* endotoxic shock, we demonstrated that MVs^nmMLCK-/-^ activated pro-resolving pathways by significant reduction of *in vitro* pro-inflammatory secretome and *in vivo* improvement of endothelial function and vascular reactivity.

## Materials and Methods

### Animals

This study was performed in male C57BL/6 of 8 week-old nmMLCK^+/+^ (Wild type) and nmMLCK^-/-^ (knockout) mice generated as previously described by selective exon targeting (Wainwright et al., 2003). All animal care and treatment procedures were performed in accordance with institutional guidelines. Protocols were approved by the French Animal Care Committee in accordance with European regulations (CEEA.PdL2012.94).

### Circulating MV Isolation

Circulating MVs were isolated from peripheral blood by successive centrifugations. Blood was centrifuged to obtain PFP as described (Mostefai et al., 2008). Sixty microliters of PFP were stored at -80°C for MV phenotyping. In order to pellet MVs for *in vitro* and *in vivo* studies, MVs were concentrated from PFP by centrifugation (21,000 × *g*, 45 min), suspended in 0.9% NaCl and stored at 4°C. Size of MVs was determined by using Malvern Zetasizer (Malvern, UK). For this, 2 μl of concentrated MVs were diluted with 300 μl of 0.9% NaCl and proceed to the analysis. Size was comprised between 100–800 nm which corresponds to the interval used in the definition of MVs. The MV concentration used for *in vitro* and *in vivo* studies corresponds to the circulating levels found in mice, whereas the control conditions correspond to treatment by vehicle (0.9% NaCl). MV levels were comprised between 811–3461 MV/μl of plasma and 1174–4581 MV/μl of plasma for nmMLCK^+/+^ and nmMLCK^-/-^, respectively.

### Characterization of MV Phenotype

Regions corresponding to total MVs were identified in forward scatter (FSC) and side scatter (SSC) intensity dot plot representation set at logarithmic gain, depending on their diameter (0.1–1 μm) by using calibrated beads (Flow Cytometry Sub-micron Particle Size Reference Kit, Molecular Probes, Eugene, OR). MV subpopulations were discriminated in PFP according to the expression of membrane-specific antigens by flow cytometry. MVs derived from platelets, erythrocytes, leukocytes, and endothelial cells were labeled using 1 μg/ml CD61-FITC (clone 2C9.G2), TER119-FITC (clone TER-119), anti-CD45-PC5 (clone 30-F11), anti-CD54-FITC (clone YN1/1.7.4) antibodies, respectively (Biolegend, London, UK). Anti-CD133-FITC (clone 13A4) and Sca1-PC7 (clone D7) antibodies were used to identify progenitor-derived MVs (Biolegend). Irrelevant mouse immunoglobulin (Ig)G was used as an isotype-matched negative control for each sample. After 45 min of incubation, Flow-count beads (8 μl) were added to samples (8 μl) to measure MV concentration. Annexin-V (BioVision, Milpitas, CA, USA) binding was used to label phosphatidylserine using binding buffer as indicated in the manufacturer’s protocol. Ca^2+^-free buffer was used to negative control of annexin-V binding. Samples were analyzed in a flow cytometer 500 MPL System (Beckman Coulter, Villepinte, France).

### Isolation of Aortic Endothelial Cells

Primary endothelial cells were isolated from mouse aorta (AoECs) as previously described (Kobayashi et al., 2005; Tual-Chalot et al., 2010). MVs^nmMLCK+/+^ or MVs^nmMLCK-/-^ have been taken from mice that have not been treated with LPS. Cells were treated for 24 h with MVs at circulating levels detected in the plasma of mice, in the absence or presence of LPS (Sigma-Aldrich, St Quentin Fallavier, France; 10 μg/ml; Recoquillon et al., 2015).

### Cytokine Production

Cytokine production was evaluated by Ray Bio Mouse Cytokine Antibody Array 3 kit (Supplementary Figure 1A; Ray Biotech, Atlanta, GA, USA). Production of interleukin (IL)-6 and monocyte chemoattractant protein-1 (MCP-1) was evaluated by mouse ELISA (Ray Biotech).

### Staining and Imaging of Aorta Wall by Confocal Microscopy

Wild type mice were intravenously injected by MVs^nmMLCK+/+^ or MVs^nmMLCK-/-^. After 20 h, mice were intraperitoneally injected with LPS (40 mg/kg) for 4 h. Then, mice were sacrificed and thoracic aorta was isolated. In another set of experiments, aortic rings from nmMLCK^+/+^ mice were treated *ex vivo* with MVs^nmMLCK+/+^, MVs^nmMLCK-/-^, LPS (10 μg/ml), and LPS with MVs^nmMLCK+/+^ or MVs^nmMLCK-/-^, for 24 h in Dulbecco’s Modified Eagle’s medium (DMEM), 20% fetal bovine serum (FBS), 1% antibiotics. Vessels were frozen and cut in 10 μm sections. After fixation, tissue sections were incubated overnight (4°C) with anti-iNOS (BD Biosciences, San José, CA, USA), anti-nitrotyrosine (clone 1A6, Millipore, Billerica, MA, USA), and anti-macrophage marker (F4/80; Biolegend) antibodies. After washes, aorta rings were incubated 1 h at room temperature with Alexa fluor 488-labeled antibody (Interchim, Montluçon, France). *In situ* production of superoxide anion was evaluated by the fluorescent dye DHE (DHE, 3 μM, 30 min; Sigma-Aldrich). After washes, sections were mounted on glass slides and visualized with a confocal microscopy (CLMS 700, Zeiss, ZEN software).

### Vascular Reactivity

After different treatments, mice were sacrificed and thoracic aorta were cleaned and cut into rings (1.5–2 mm length). Aortic rings were mounted on a wire myograph to record isometrical mechanical activity, as previously described (Tual-Chalot et al., 2010; Leonetti et al., 2013). Arteries were precontracted to 80% of maximal contraction with the thromboxane A2 analog 9,11-Dideoxy-9a,11a-methanoepoxy prostaglandin F2α (U-46619, Merck Chemicals, Nottingham, UK) and endothelium-dependent relaxation was assessed by cumulative addition of acetylcholine (1 nM–10 μM). Neither MVs^nmMLCK+/+^ nor MVs^nmMLCK-/-^ did induce differences in the levels of pre-contraction induced by U-46619, which attest the absence of LPS contamination on MV preparation. Also, vascular contraction was evaluated by cumulative application of 5-HT (5-HT, 0.1 nM–10 μM; Sigma Aldrich) to vessels with functional endothelium (Ralay Ranaivo et al., 2007).

### Nitric Oxide (NO) Assay by Griess Reaction

AoECs were treated with MVs^nmMLCK+/+^, MVs^nmMLCK-/-^, LPS (10 μg/ml), or the combination LPS with MVs for 24 h. The culture medium was collected and mixed with Griess reagent (Sigma-Aldrich) and nitrate reductase. Sodium nitrite standards were used to normalize the assay.

### NO Spin Trapping and Electronic Paramagnetic Resonance (EPR) Studies

The detection of NO production was performed using the technique with Fe^2+^ diethyldithiocarbamate (DETC, Sigma Aldrich) as spin trap. Isolated heart and liver from mice injected with LPS, LPS with MVs^nmMLCK+/+^ or MVs^nmMLCK-/-^, and vehicle were incubated for 45 min in Krebs-Hepes buffer [bovine serum albumin (20.5 g/l), CaCl_2_ (3 mM) and L-arginine (0.8 mM); Sigma-Aldrich] and after treated with 250 μl of colloid Fe(DETC)_2_ and incubated at 37°C for 45 min (Ralay Ranaivo et al., 2007; Leonetti et al., 2013). The organs were immediately frozen in plastic tubes. NO measurements were performed on a tabletop x-band spectrometer miniscope (MS200; Magnettech, Berlin, Germany). Values are expressed as amplitude of signal per weight of dried tissue.

### Data Analysis

Data were analyzed using GrapPad Prism Software (GrapPad Software, San Diego, CA, USA). Data are expressed as mean ± SEM, and n represents the number of mice. Statistical analyses were performed with non-parametric Mann-Whitney tests or two-way analysis of variance for repeated measures and subsequent Bonferroni *post hoc* tests. *P* < 0.05 was considered to be statistically significant.

## Results

### Deletion of nmMLCK Protects against the Increase in Circulating MVs as Well as Aortic Oxidative and Nitrative Stress and Macrophage Infiltration Induced by LPS

**FIGURE 1:**
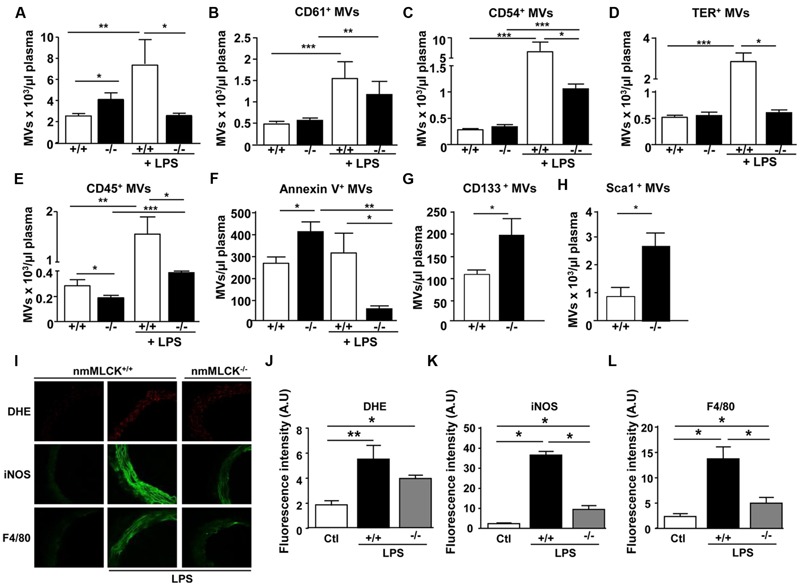
**Deletion of non-muscular myosin light chain kinase (nmMLCK) prevents changes on MV levels induced by LPS.** Circulating MVs collected from blood were incubated with antibodies to discriminate MV subpopulations according to the expression of membrane specific antigens by flow cytometer. Histograms show total circulating MVs **(A)**, platelet-(CD61^+^) **(B)**, endothelial cell- (CD54^+^) **(C)**, erythrocyte-(TER^+^) **(D)**, leukocyte-(CD45^+^) **(E)**, procoagulant-(annexin V^+^) **(F)**, and progenitor cell- (CD133^+^, Sca1^+^) **(G,H)** MVs from nmMLCK^+/+^ and nmMLCK^-/-^ mice treated intraperitoneally or not with LPS (40 mg/kg) for 4 h. Values are expressed as events per microliters and the data represent the mean ± SEM (*n* = 16–21) ^∗^*P* < 0.05, ^∗∗^*P* < 0.01, ^∗∗∗^*P* < 0.001. **(I)** Confocal image staining of superoxide anion production by DHE (red), iNOS (green), and macrophage infiltration (F4/80 green) in thoracic aorta from nmMLCK^+/+^ and nmMLCK^-/-^ mice injected intraperitoneally by LPS (40 mg/kg) for 4 h. Histograms show fluorescence intensity of aorta DHE staining **(J)**, iNOS **(K)**, and F4/80 **(L)**, assessed by Image J. Data are expressed in arbitrary units (A.U.) of fluorescence intensity and represent the mean ± SEM (*n* = 3–5) ^∗^*P* < 0.05, ^∗∗^*P* < 0.01.

Deletion of nmMLCK slightly, but significantly, increased circulating MVs compared to wild type (**Figure 1A**). This was associated with slight increase of procoagulant (annexin V^+^) but a significant decrease of leukocytes (CD45^+^)-MVs without changes in both platelets (CD61^+^)-, endothelial cells (CD54^+^)-, and erythrocytes (TER^+^)-MVs (**Figures 1A–F**). In addition, MVs from progenitor cells were significantly increased (**Figures 1G,H**). Treatment with LPS induced an increase in levels of circulating MVs in wild type mice especially those derived from platelets, endothelial cells, erythrocytes, and leukocytes without affecting procoagulant MVs (**Figures 1A–F**). Interestingly, LPS failed to enhance circulating MVs in nmMLCK deficient mice. Moreover, the increase in levels of platelet-, endothelial-, and leukocyte-derived MVs was less pronounced in nmMLCK^-/-^ mice, whereas no changes were observed in TER^+^-derived MVs. In accordance with our previous work (Ralay Ranaivo et al., 2007), *in vivo* LPS treatment induced increased DHE, iNOS, and F4/80 labeling in the aortic wall from wild type whereas a slight increase was induced in vessels from nmMLCK^-/-^ mice (**Figures 1I–L**). These results indicate that nmMLCK deletion protects against oxidative and nitrative stress as well as macrophage infiltration induced by LPS (**Figures 1G–J**).

### MVs from nmMLCK^-/-^ Mice Have No Effects on Mouse AoECs or Aorta with Respect to Inflammatory Cytokine Release, Oxidative, and Nitrative Stresses as Well as Endothelial Function

**FIGURE 2:**
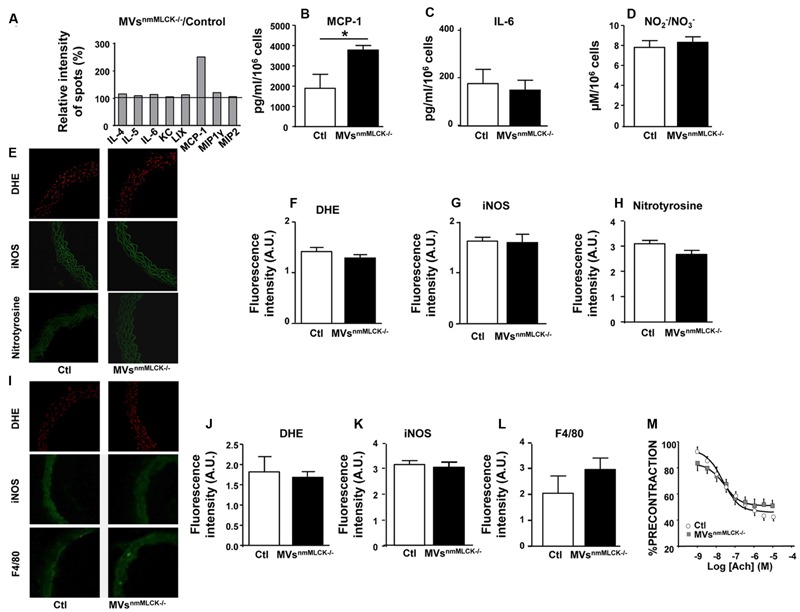
**Lack of effects of circulating MVs from non-muscular myosin light chain kinase (nmMLCK)-deficient mice.** Primary endothelial cells isolated from mouse aorta (AoECs) of nmMLCK^+/+^ mice were treated for 24 h with MVs^nmMLCK-/-^. Cytokine production was evaluated by Ray Bio Mouse Cytokine Antibody Array 3 kit (Supplementary Figure 1A). **(A)** Histograms show the cytokine ratio between either MVs^nmMLCK-/-^-treated versus non-treated cells (Control). Black line represents cytokine production of non-treated cells. Results are expressed as a percentage of relative intensity of spots (%). Data represent the mean of *n* = 3. Quantification of MCP1 **(B)** and IL-6 **(C)** secretion by ELISA assay. Data represent the mean of *n* = 3, ^∗^*P* < 0.05. **(D)** Quantification of ${\mathrm{NO}}_{2}^{–}$/${\mathrm{NO}}_{3}^{–}$ (nitrite/nitrate) from the supernatant of AoECs previously treated by MVs^nmMLCK-/-^, using Griess assay. **(E–H)** Confocal image staining of superoxide anion production by DHE (red), iNOS (green), and nitrotyrosine expression (green) in mouse wild type aorta exposed 24 h *ex vivo* to either saline salt solution (Ctl) or MVs^nmMLCK-/-^. Aorta was imaged using confocal microscope. **(F–H)** Histograms show fluorescence intensity of aortic DHE staining **(F)**, iNOS **(G)** and nitrotyrosine **(H)** assessed by Image J. Background fluorescence intensity was subtracted using unstained aortas. Data are expressed in arbitrary units (A.U.) of fluorescence intensity and represent the mean ± SEM (*n* = 3–5). **(I–L)** Confocal image staining of DHE (red), iNOS expression (green), and macrophage infiltration (F4/80 green) in aorta from wild type mice injected intravenously with saline salt solution (Ctl) or MVs^nmMLCK-/-^ for 24 h. Histograms show fluorescence intensity of aortic DHE staining **(J)**, iNOS **(K)** and F4/80 **(L)**, assessed by Image J. Data expressed in A.U. of fluorescence intensity and represent the mean ± SEM (*n* = 3–5). **(M)** Endothelium-dependent relaxation induced by acetylcholine (Ach) was evaluated by myography on aortic rings from wild type mice treated intravenously with MVs^nmMLCK-/-^ at their circulating concentration. The relaxation was expressed as a percentage of precontraction level. Statistical analyses were performed using two-way ANOVA test. Data represent the mean of *n* = 3.

In order to decipher whether MVs from nmMLCK^-/-^ mice participate in mechanism of protection against LPS treatment, we first examined the effects that these MVs on their own. Thus, we analyzed by antibody array screening the effect of MVs^nmMLCK-/-^ on inflammatory cytokines on mouse AoECs (**Figure 2A**). Treatment with MVs^nmMLCK-/-^ enhanced the production of MCP-1 by AoECs without affecting secretion of other cytokines such as IL-4, IL-5, or IL-6 (**Figures 2A–C**). In addition, no significant changes on nitrite/nitrate production reflecting nitrative stress were observed in AoECs after treatment by MVs^nmMLCK-/-^ (**Figure 2D**). When aortic rings from wild type mice were *in vitro* incubated with MVs^nmMLCK-/-^ no significant changes in inflammatory markers (DHE, iNOS) or nitration of proteins were observed (**Figures 2E–H**). Also, when mice were injected with MVs^nmMLCK-/-^, no effects were detected concerning oxidative and nitrative stresses or macrophage infiltration (**Figures 2I–L**).

Aortic rings from nmMLCK^+/+^ mice relaxed in response to acetylcholine up to a maximum of relaxation at 10 μM (60% of relaxation; **Figure 2M**). Treatment with MVs^nmMLCK-/-^ did not modify the relaxation induced by acetylcholine. Altogether, these results suggest that MVs^nmMLCK-/-^ by themselves do not induce inflammation nor modify vascular function.

### MVs^nmMLCK-/-^ Protect against the Effects of LPS-Induced Inflammation by Increasing Anti-Inflammatory and Reducing Pro-Inflammatory Secretome in AoECs

First, the cytokine contents of MVs from wild type and nmMLCK-deficient mice were assessed. No significant differences on the cytokine content were observed between both types of MVs (Supplementary Figure 1B).

**FIGURE 3:**
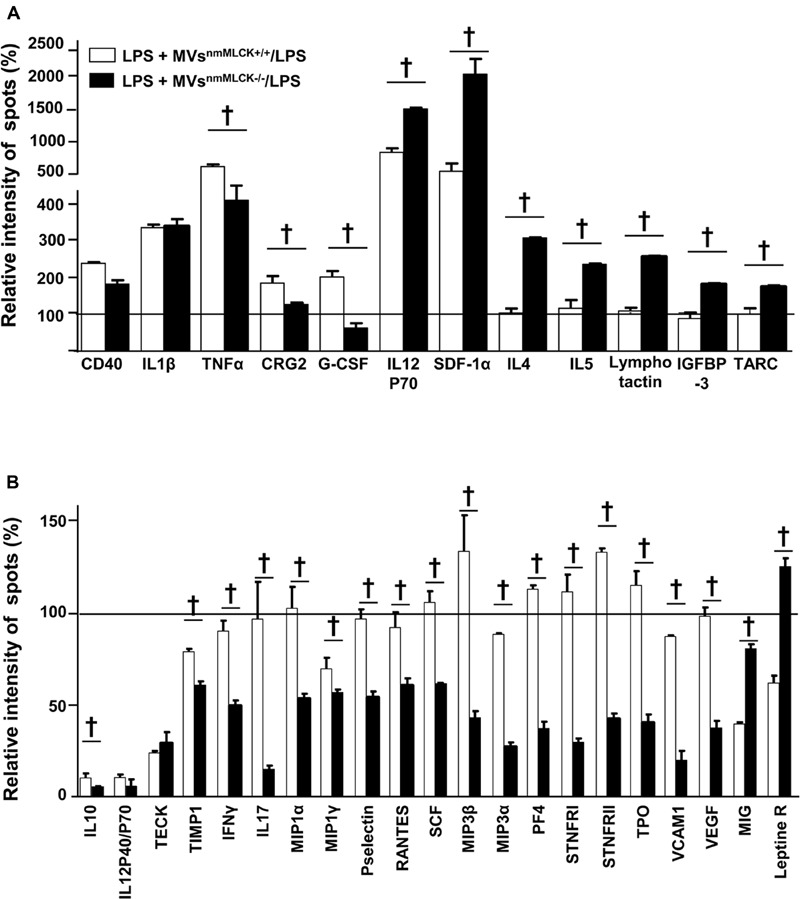
**Microvesicles from non-muscular myosin light chain kinase-deficient mice (MVs^nmMLCK-/-^) increase anti- inflammatory and reduce pro-inflammatory secretome of aortic endothelial cells (AoECs).** Primary AoECs from wild type mice were treated for 24 h with MVs^nmMLCK+/+^ or MVs^nmMLCK-/-^ in the absence or in the presence of LPS (10 μg/ml). Cytokine production was evaluated by Ray Bio Mouse Cytokine Antibody Array 3 kit (Supplementary Figure 1A for more details). Data represent the mean ± SEM (*n* = 3–5). **(A,B)** Histograms show the cytokine ratio between either LPS+MVs^nmMLCK+/+^-treated versus LPS-treated cells or LPS+MVs^nmMLCK-/-^-treated versus LPS-treated cells. Black line represents no change in cytokine production between treatments. Results are expressed as a percentage of relative intensity of spots (%). Data represent the mean of three experiment (*n* = 3–5). ^†^*P* < 0.05.

In the presence of LPS, MVs^nmMLCK+/+^ increased the production of pro-inflammatory cytokines such as CD40, IL-1β, tumor necrosis factor alpha (TNFα), IL-12P70 active form of IL-12 cytokine (observed also by decreased ratio IL12P40/P70, IL12P40 antagonist form of IL12P70), and SDF (SDF-1α; **Figures 3A,B**). Furthermore, MVs^nmMLCK+/+^ reduced anti-inflammatory cytokine (IL-10), and pro-inflammatory chemokines (TECK, MIG; **Figure 3B**). In contrast, MVs^nmMLCK-/-^ induced a lower increase of TNFα, CRG2, and G-CSF and a higher enhancement of IL-12P70 and SDF-1α than MVs^nmMLCK+/+^ treatment (**Figure 3B**). Interestingly, MVs^nmMLCK-/-^ enhanced the release of anti-inflammatory cytokines (IL-4 and IL-5), IGFBP-3, and lymphocyte chemokines (lymphotactin and TARC; **Figure 3B**). Moreover, MVs^nmMLCK-/-^ decreased several pro-inflammatory cytokines (TIMP1, IFNγ, IL-17, MIP family, P-selectin, Rantes, VCAM1; **Figure 3B**). These results show that the protective effect of MVs^nmMLCK-/-^ against the deleterious effects of LPS was associated to their ability both to increase anti- inflammatory and to reduce pro-inflammatory secretome in AoECs.

### MVs^nmMLCK-/-^ Prevent Oxidative and Nitrative Stresses Induced by *In vitro* Treatment with LPS on Mouse Aorta

**FIGURE 4:**
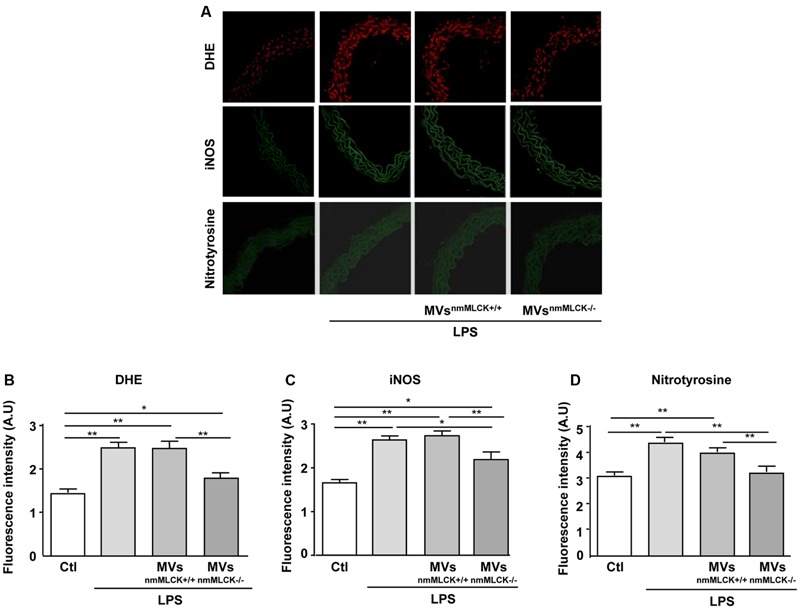
**Microvesicles from non-muscular myosin light chain kinase-deficient mice (MVs^nmMLCK-/-^) reduce the LPS-evoked oxidative and nitrative stresses in mouse aorta. (A)** Confocal image staining of DHE (red), iNOS (green), and nitrotyrosine expression (green) in mouse wild type aorta exposed 24 h *ex vivo* to saline salt solution, LPS alone (10 μg/ml), LPS + MVs^nmMLCK+/+^ or LPS + MVs^nmMLCK-/-^. Aorta was imaged using confocal microscope. **(B–D)** Histograms show fluorescence intensity of aorta DHE staining **(B)**, iNOS **(C)**, and nitrotyrosine **(D)** assessed by Image J. Data represent arbitrary units (A.U.) of the mean ± SEM (*n* = 3–5). ^∗^*P* < 0.05, ^∗∗^*P* < 0.01.

As expected, LPS incubation significantly increased $O_{2}^{–}$ production, iNOS expression and nitration of tyrosine in aortas from wild type mice (**Figures 4A–D**). In the presence of LPS, MVs^nmMLCK+/+^ did not modify the DHE labeling, iNOS expression, and nitrotyrosine levels when compared to the LPS alone (**Figures 4A–D**). Interestingly, MVs^nmMLCK-/-^ prevented the effect of LPS to induce $O_{2}^{–}$ production and tyrosine nitration and partially reduced iNOS overexpression in the aorta (**Figures 4A–D**). These responses were reinforced by the fact that LPS was not able to increase nitrite/nitrate and nitration of proteins in the presence of MVs^nmMLCK-/-^ in AoECs (not shown).

### MVs^nmMLCK-/-^ Correct *Ex vivo* Deleterious Effect of LPS on Vascular Function and *In vivo* NO Production in Heart and Liver

**FIGURE 5:**
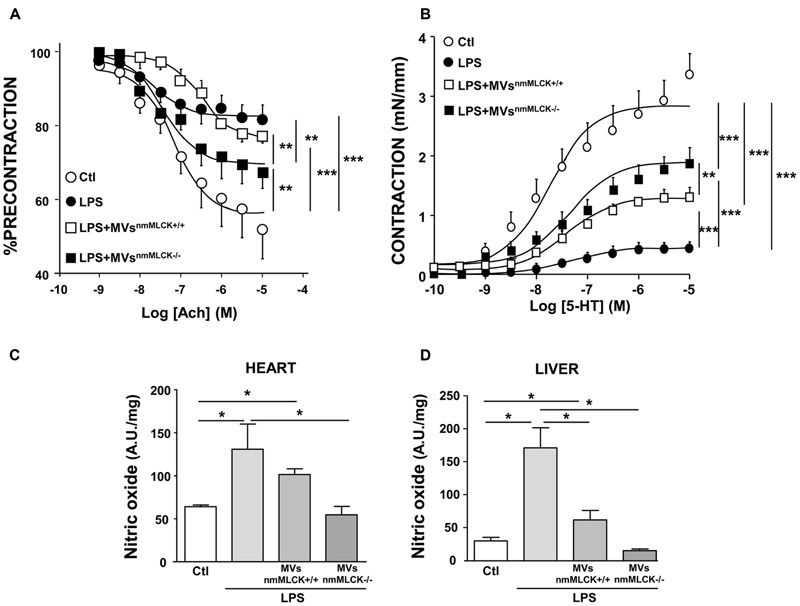
**Microvesicles from non-muscular myosin light chain kinase-deficient mice (MVs^nmMLCK-/-^) improve the vascular function induced by LPS *in vivo* and the nitrative stress in heart and liver.** Wild type mice were injected intravenously with saline salt solution (Ctl), MVs^nmMLCK+/+^, or MVs^nmMLCK-/-^. After 20 h, mice were injected intraperitoneally with LPS (40 mg/Kg) for 4 h. **(A)** Endothelium-dependent relaxation induced by acetylcholine (Ach) was evaluated by myography. The relaxation was expressed as a percentage of precontraction level. **(B)** Concentration-effect curves in response to 5-HT in aortic rings isolated from mice. Contraction are expressed in mN/mm. **(C,D)** NO production assessed by the amplitude of NO-Fe(DETC)_2_ complex signal in unit/weight in heart **(C)** and liver **(D)** from mice. Data represent arbitrary units (A.U.) of the mean ± SEM/mg of tissue weight (*n* = 3–5). ^∗^*P* < 0.05, ^∗∗^*P* < 0.01, ^∗∗∗^*P* < 0.001.

As expected, treatment with LPS induced significant reduction of the maximal of relaxation (∼80% of inhibition) compared to vessels taken from vehicle-injected animals, which traduces endothelial dysfunction (**Figure 5A**). MVs^nmMLCK+/+^ injection did not modify the LPS-induced impairment on endothelial relaxation. Interestingly, MVs^nmMLCK-/-^ partially corrected the effect of LPS on the endothelium-dependent relaxation induced by acetylcholine. In addition, hyporeactivity induced by LPS was partially corrected by both types of MVs; however, the effects of MVs^nmMLCK-/-^ were significantly higher than those of MVs^nmMLCK+/+^ (**Figure 5B**).

Also, whereas MVs^nmMLCK+/+^ had not effect or partially reduced LPS-evoked NO production in heart and liver, respectively, MVs^nmMLCK-/-^ abolished the increase in NO production induced by LPS treatment in these tissues (**Figures 5C,D**). Together, these results suggest that deletion of nmMLCK leads to protective effects of MVs to correct LPS inflammation.

## Discussion

Here, we show that nmMLCK-deficient mice display a resistance to LPS-induced increase in circulating MVs and vascular oxidative/nitrative stress and macrophage infiltration. Most importantly, we provide evidence that MVs issued from nmMLCK-deficient mice prevent the inflammatory effects of LPS with concomitant increase of anti- inflammatory and reduction of pro-inflammatory secretome from endothelial cells. In addition, MVs^nmMLCK-/-^ lead to a reduced efficacy of LPS to increase aortic oxidative and nitrative stress as well as macrophage infiltration in the aorta. Moreover, MVs^nmMLCK-/-^ correct *ex vivo* deleterious effect of LPS on endothelial function, vascular reactivity, and *in vivo* tissular overproduction of NO. Altogether, these findings provide evidence that nmMLCK deletion generates circulating MVs displaying protective effects by activating pro-resolving anti-inflammatory pathways allowing the effective down-regulation of oxidative and nitrative stresses associated with endotoxic shock.

Sepsis in general, and endotoxic shock in particular, is characterized, at the vascular level, by an increase of inflammation resulting from an enhanced secretion of cytokines and chemokines, an exacerbated production of reactive oxygen species, and the induction of iNOS leading to enhanced NO production which accounts for vascular hyporeactivity and endothelial dysfunction. These events favor macrophage infiltration on the vessel wall and finally lead to organ failure (for review see Sherwood and Toliver-Kinsky, 2004). We have previously reported that, in several models of endotoxemia (LPS and cecal ligation puncture), nmMLCK^-/-^ mice are protected against nitrative and oxidative stresses at the level of the vascular wall and the subsequent increase of survival when compared to wild type mice (Ralay Ranaivo et al., 2007). Here, we show that LPS treatment increase MV formation in wild type mice and, interestingly, nmMLCK^-/-^ mice are protected against this increase. Enhanced levels of circulating MVs from platelets, granulocytes, and endothelial cells have been described in patients with meningococcal septicemia, and septic shock (Nieuwland et al., 2000; Fujimi et al., 2002; Mostefai et al., 2008). MVs participate in organ dysfunction observed in septic shock patients (Mastronardi et al., 2011) despite the reported correlation between increased circulating MPs and better survival rate among patients in the early phase of septic shock (Soriano et al., 2005). Also, inoculation of septic MVs in healthy rats reproduced hemodynamic, septic inflammatory patterns, associated with oxidative and nitrative stresses (Mortaza et al., 2009). Thus, the prevention of increase of circulating MVs by nmMLCK deletion may participate in the correction of the deleterious effect of LPS at the level of the vascular wall.

On the other hand, the present results suggest that nmMLCK is probably implicated on the process of MV formation. It is well known that cytoskeleton disorganization represents an essential step in the process of MV generation, and at this level, nmMLCK may interact with other cytoskeleton proteins and contribute to the MV formation (Owens and Mackman, 2011). Indeed, several studies have shown that inhibition of MLCK with ML-7 reduces MV production suggesting that MLCK participates in the process of membrane blebbing leading to MV formation and/or release (Mills et al., 1998; Muralidharan-Chari et al., 2009; Midura et al., 2016). It has been shown that activation of MLCK by Ca^2+^/calmodulin or by tyrosine kinase phosphorylated regulatory MLC-2 resulting in a change in the myosin tertiary structure favoring contractile movement against actin at the necks of MVs, facilitating their release into the extracellular space (Muralidharan-Chari et al., 2009). At the opposite, recent data show that ML-7 increases the number of blebs/min/cell suggesting that MLCK controls membrane dynamics (Barfod et al., 2011), that is in agreement with the present results demonstrating that the deletion of nmMLCK increased the number of circulating MVs. The increase in the number of circulating MVs was not probably due to their decreased clearance on nmMLCK^-/-^ mice because alterations on *in vitro* production of platelet-derived MVs from nmMLCK^-/-^ mice were also observed (data not shown).

In the present study, we showed that MVs may modulate the secretion of inflammatory mediators from endothelial cells by LPS. Modification of the secretory capacity of endothelial cells alters the function of these cells leading, in the long run, to the development of cardiovascular diseases (Libby, 2000). Whereas MVs from nmMLCK^+/+^ mice increased the release of pro-inflammatory versus anti-inflammatory cytokines, MVs from nmMLCK^-/-^ mice induced opposite effects suggesting that both types of MVs are able to regulate cytokine production in endothelial cells but they activate different pathways in order to produce different cytokines. It is accepted that MVs can harbor cytokines to be transfer into the target cells. Thus, it has been shown that human monocytes treated by LPS were able to release MVs carrying bioactive IL-1β and transcripts for pro-inflammatory cytokines such as TNF, IL-6, and IL-8 (MacKenzie et al., 2001; Wen et al., 2014). However, in the present study we have described that MVs from both wild type and nmMLCK knock out mice harbor identical cytokine contents. Other authors have reported that MVs released by human endothelial cells and monocytes after TNF-α stimulation up-regulated podocyte production of pro-inflammatory MCP-1 and IL-6 (Eyre et al., 2011). Interestingly, under pro-inflammatory conditions such as LPS infusion, injection on systemic circulation of MVs generated *in vitro* from human endothelial cells increased levels of IL-1β and TNF-α, suggesting that MVs prime injury-associated inflammation in mice (Buesing et al., 2011). In contrast, other types of MVs possessed anti-inflammatory and beneficial effects. Activated human neutrophils released MVs were able to reduce inflammatory response mediated by macrophages exposed to LPS (Gasser and Schifferli, 2004) by mechanism related to annexin A1 (Dalli et al., 2008). Similarly, MVs enriched in alpha-2-macroglobulin preserved neutrophil chemotactic responses in the presence of LPS and consequently activated pro-resolving pathways (Dalli et al., 2014). Here, the protective effects of MVs from nmMLCK-/- mice may be related with a different composition of MVs. Indeed, as described above, deletion of nmMLCK can affect not only the process of MV formation but also their content. In this respect, nmMLCK has been reported to contain amino acid sequence motifs associated with subcellular targeting or protein-protein interactions in the proteome (Lin et al., 1999; Smith et al., 2002). This domain of the enzyme plays a role as a cellular organizer, providing integration among diverse protein including cytoskeletal proteins (Kudryashov et al., 2004) and NF-κB (Recoquillon et al., 2015). Congruent with that hypothesis, MLCK activity has been shown to drive TNFα-dependent NF-κB activation and amplification. Thus, cells from nmMLCK-deficient mice might not able to activate this pathway and modify the generated MVs to be less inflammatory.

Interestingly, we demonstrate that MVs^nmMLCK-/-^ partially corrected, at the level of vascular wall, inflammatory responses elicited by LPS in *in vitro* and in *in vivo* conditions. Indeed, MVs^nmMLCK-/-^ reduced iNOS expression, superoxide anion production, and macrophage infiltration into the vascular wall. At a consequence, MVs^nmMLCK-/-^ restore vascular function after LPS treatment since endothelium-dependent relaxation and vascular contraction were improved. Furthermore, nitrative stress in vital organs such as heart and liver were abolished, suggesting that MVs^nmMLCK-/-^ act, not only at the vascular level, but also in other organs essential to maintain life. Our previous study shows that nmMLCK is involved in lethal complications as well as in the vascular reactivity changes associated with endotoxic shock (Ralay Ranaivo et al., 2007). nmMLCK is linked to LPS-induced up-regulation of NF-κB and increased oxidative and nitrative stresses (Recoquillon et al., 2015). The present study and our former reports underscore that inoculation of MVs from nmMLCK-deficient mice into wild type mice or the use of nmMLCK-deficient mice display similar protective effect in the experimental model of endotoxic shock used in terms of oxidative/nitrative stress, vascular and tissular dysfunction. We further demonstrate that nmMLCK plays a role in the deleterious messages carried by MVs leading to increased susceptibility to sepsis and its consequences in both cardiovascular and systemic injuries.

### Limitations of the Study

Up to now, nmMLCK has been involved in the regulation of the endothelial cell permeability by regulating cytoskeletal rearrangement through the ATP-dependent interaction of actin and myosin on endothelial cells (Shen et al., 2010). Deletion of nmMLCK affects the production of MVs and probably their content. Further studies are needed to fully characterize the composition of MVs^nmMLCK-/-^, and in this way, to decipher the exact mechanism implicated in the opposite effects of MVs depending on their origin (nmMLCK wild type or knock out mice). Because cytokine content of both types of MVs is not different, we can hypothesize that MVs^nmMLCK-/-^ might carry transcription factors or miRNA presenting anti-inflammatory functions which contribute to reduce inflammatory effect of LPS on target cells.

Besides, therapy with engineered nmMLCK-deleted MVs may represent an important tool of personalized medicine since these MVs display anti-inflammatory properties. Thus, they could well represent new and promising therapeutic strategies and these results may be transposed to sepsis in humans.

## Conclusion

This study provides evidence that deletion of nmMLCK generates circulating MVs with a protective potential. They underscore that nmMLCK may represent a candidate in the regulation of pro-resolving responses in sepsis by engineered MVs.

## Author Contributions

AG and LV: Performed experiments and analyzed data; AG, LV, and MM: Interpreted data; RA and MM: Drafted of the manuscript; RA and MM: Designed the study.

## Conflict of Interest Statement

The authors declare that the research was conducted in the absence of any commercial or financial relationships that could be construed as a potential conflict of interest.

## Abbreviations

AoECs: aorta endothelial cells
DHE: dihydroethidine
iNOS: inducible nitric oxide synthase
IGFBP-3: insulin like growth factor binding protein 3
MVs: microvesicles
MVs^nmMLCK+/+^: microvesicles from wild type mice
MVs^nmMLCK-/-^: microvesicles from nmMLCK deficient mice
MLC-2: myosin light chain
nmMLCK: non-muscle myosin light chain kinase
PFP: platelet-free plasma
5-HT: serotonin
SDF-1α: stromal cell-derived factor 1 alpha
TARC: thyroid activation regulated chemokine
