# Supplementary material for: Activation of Endothelial Pro-resolving Anti-Inflammatory Pathways by Circulating Microvesicles from Non-muscular Myosin Light Chain Kinase-Deficient Mice
Source: Front Pharmacol. 2016 Sep 21;7:322. doi: 10.3389/fphar.2016.00322 (PMC5030219; doi:10.3389/fphar.2016.00322)
Supplement: Supplementary file 1 [file Image_1.PDF]

A

	A	B	C	D	E	F	G	H	I	J	K	L	M	N
1	POS	POS	NEG	NEG	BLANK	Axl	BLC	CD30 L	CD30	CD40	CRG-2	CTACK	CXCL16	Eotaxin1
2	POS	POS	NEG	NEG	BLANK	Axl	BLC	CD30 L	CD30	CD40	CRG-2	CTACK	CXCL16	Eotaxin1
3	Eotaxin 2	Fas Ligand	CX3CL1	GCSF	GM-CSF	IFN gamma	IGFBP 3	IGFBP 5	IGFBP 6	IL-1 alpha	IL-1 beta	IL-2	IL-3	IL-3 Rb
4	Eotaxin 2	Fas Ligand	CX3CL1	GCSF	GM-CSF	IFN gamma	IGFBP 3	IGFBP 5	IGFBP 6	IL-1 alpha	IL-1 beta	IL-2	IL-3	IL-3 Rb
5	IL-4	IL-5	IL-6	IL-9	IL-10	IL12 p40/70	IL-12 p70	IL-13	IL-17	KC	Leptin R	Leptin	LIX	L Selectin
6	IL-4	IL-5	IL-6	IL-9	IL-10	IL12 p40/70	IL-12 p70	IL-13	IL-17	KC	Leptin R	Leptin	LIX	L Selectin
7	Ltn/ XCL1	MCP 1	MCP-5	M-CSF	MIG	MIP-1 alpha	MIP-1 gamma	MIP-2	MIP-3 beta	MIP-3 alpha	PF-4	P Selectin	RANTES	SCF
8	Ltn/ XCL1	MCP 1	MCP-5	M-CSF	MIG	MIP-1 alpha	MIP-1 gamma	MIP-2	MIP-3 beta	MIP-3 alpha	PF-4	P Selectin	RANTES	SCF
9	SDF-1 alpha	TARC	TCA-3	TECK	TIMP-1	TNF alpha	sTNFRI	sTNFRII	TPO	VCAM-1	VEGF	BLANK	BLANK	POS
10	SDF-1 alpha	TARC	TCA-3	TECK	TIMP-1	TNF alpha	sTNFRI	sTNFRII	TPO	VCAM-1	VEGF	BLANK	BLANK	POS

B

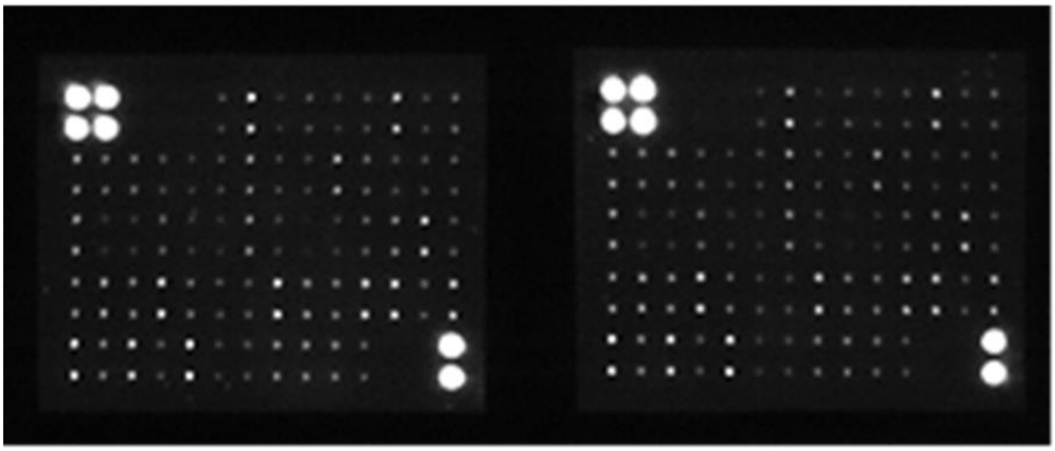

MVsnmMLCK+/+ MVsnmMLCK-/-

Supplemental Data. (A) Template showing location of antibodies for cytokines spotted onto RayBiotech cytokine antibody array kit (62 cytokines). Each spot on the membrane represents one cytokine. (B) Image of membranes of antibody array screening using for analysis of cytokines carried by MVs. No differences between cytokines carried by both types of MVs were deteted.
